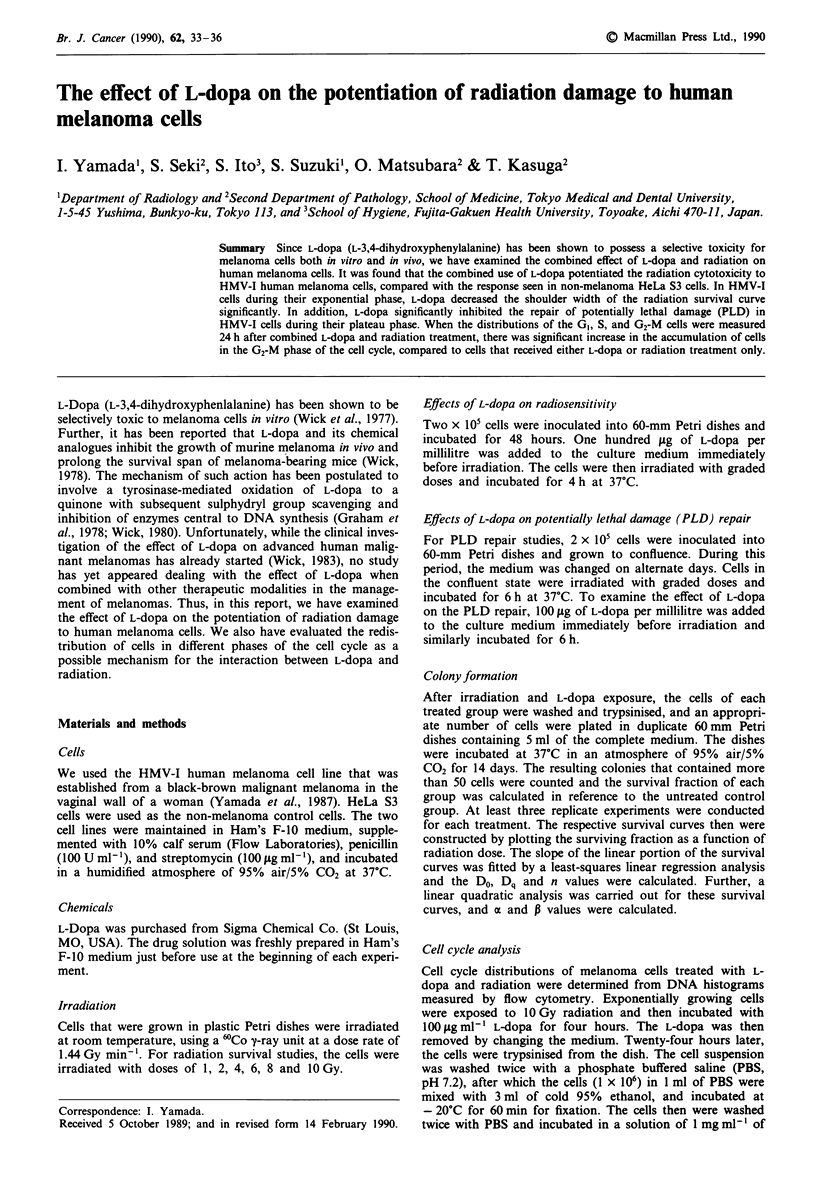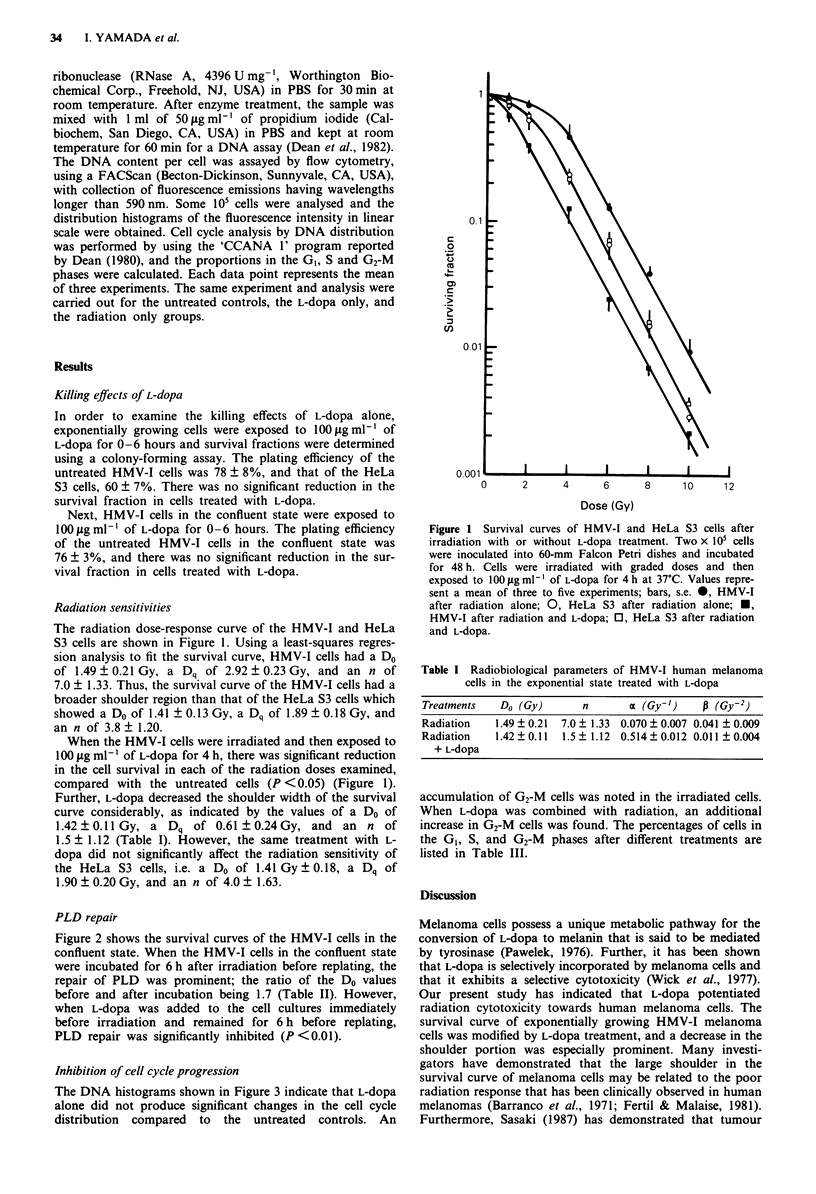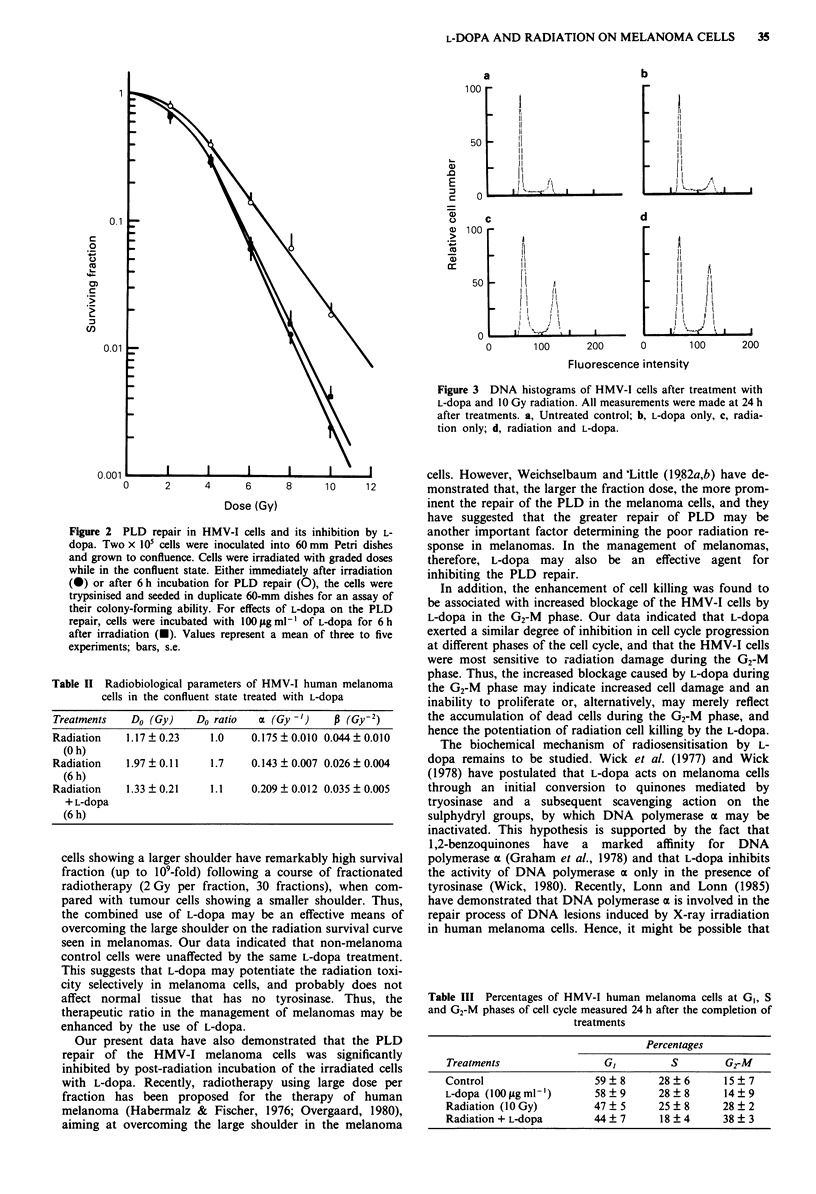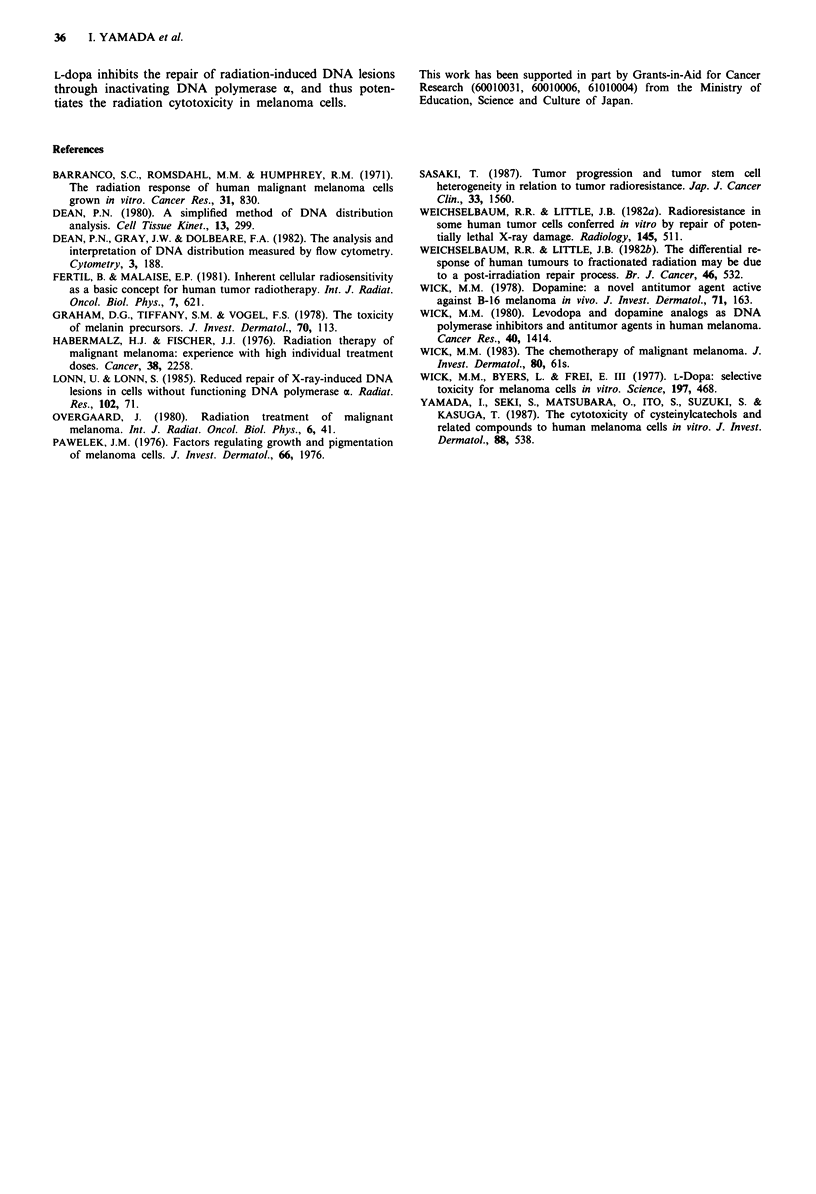# The effect of L-dopa on the potentiation of radiation damage to human melanoma cells.

**DOI:** 10.1038/bjc.1990.224

**Published:** 1990-07

**Authors:** I. Yamada, S. Seki, S. Ito, S. Suzuki, O. Matsubara, T. Kasuga

**Affiliations:** Department of Radiology, School of Medicine, Tokyo Medical and Dental University, Japan.

## Abstract

Since L-dopa (L-3,4-dihydroxyphenylalanine) has been shown to possess a selective toxicity for melanoma cells both in vitro and in vivo, we have examined the combined effect of L-dopa and radiation on human melanoma cells. It was found that the combined use of L-dopa potentiated the radiation cytotoxicity to HMV-I human melanoma cells, compared with the response seen in non-melanoma HeLa S3 cells. In HMV-I cells during their exponential phase, L-dopa decreased the shoulder width of the radiation survival curve significantly. In addition, L-dopa significantly inhibited the repair of potentially lethal damage (PLD) in HMV-I cells during their plateau phase. When the distributions of the G1, S, and G2-M cells were measured 24 h after combined L-dopa and radiation treatment, there was significant increase in the accumulation of cells in the G2-M phase of the cell cycle, compared to cells that received either L-dopa or radiation treatment only.


*Br. J. Cancer* (1990), **62**, 33–36 © Macmillan Press Ltd., 1990

### The effect of L-dopa on the potentiation of radiation damage to human melanoma cells

I. Yamada[1], S. Seki[2], S. Ito[3], S. Suzuki[1], O. Matsubara[2] & T. Kasuga[2]

[1]*Department of Radiology and* [2]*Second Department of Pathology, School of Medicine, Tokyo Medical and Dental University, 1-5-45 Yushima, Bunkyo-ku, Tokyo 113, and* [3]*School of Hygiene, Fujita-Gakuen Health University, Toyoake, Aichi 470-11, Japan.*

**Summary** Since L-dopa (L-3,4-dihydroxyphenylalanine) has been shown to possess a selective toxicity for melanoma cells both *in vitro* and *in vivo*, we have examined the combined effect of L-dopa and radiation on human melanoma cells. It was found that the combined use of L-dopa potentiated the radiation cytotoxicity to HMV-I human melanoma cells, compared with the response seen in non-melanoma HeLa S3 cells. In HMV-I cells during their exponential phase, L-dopa decreased the shoulder width of the radiation survival curve significantly. In addition, L-dopa significantly inhibited the repair of potentially lethal damage (PLD) in HMV-I cells during their plateau phase. When the distributions of the $G_1$, S, and $G_2$-M cells were measured 24 h after combined L-dopa and radiation treatment, there was significant increase in the accumulation of cells in the $G_2$-M phase of the cell cycle, compared to cells that received either L-dopa or radiation treatment only.

L-Dopa (L-3,4-dihydroxyphenlalanine) has been shown to be selectively toxic to melanoma cells *in vitro* (Wick *et al.*, 1977). Further, it has been reported that L-dopa and its chemical analogues inhibit the growth of murine melanoma *in vivo* and prolong the survival span of melanoma-bearing mice (Wick, 1978). The mechanism of such action has been postulated to involve a tyrosinase-mediated oxidation of L-dopa to a quinone with subsequent sulphydryl group scavenging and inhibition of enzymes central to DNA synthesis (Graham *et al.*, 1978; Wick, 1980). Unfortunately, while the clinical investigation of the effect of L-dopa on advanced human malignant melanomas has already started (Wick, 1983), no study has yet appeared dealing with the effect of L-dopa when combined with other therapeutic modalities in the management of melanomas. Thus, in this report, we have examined the effect of L-dopa on the potentiation of radiation damage to human melanoma cells. We also have evaluated the redistribution of cells in different phases of the cell cycle as a possible mechanism for the interaction between L-dopa and radiation.

### Materials and methods

### *Cells*

We used the HMV-I human melanoma cell line that was established from a black-brown malignant melanoma in the vaginal wall of a woman (Yamada *et al.*, 1987). HeLa S3 cells were used as the non-melanoma control cells. The two cell lines were maintained in Ham's F-10 medium, supplemented with 10% calf serum (Flow Laboratories), penicillin (100 U $ml^{-1}$), and streptomycin (100 $\mu g\ ml^{-1}$), and incubated in a humidified atmosphere of 95% air/5% $CO_2$ at 37°C.

### *Chemicals*

L-Dopa was purchased from Sigma Chemical Co. (St Louis, MO, USA). The drug solution was freshly prepared in Ham's F-10 medium just before use at the beginning of each experiment.

### *Irradiation*

Cells that were grown in plastic Petri dishes were irradiated at room temperature, using a $^{60}Co$ $\gamma$-ray unit at a dose rate of 1.44 Gy $min^{-1}$. For radiation survival studies, the cells were irradiated with doses of 1, 2, 4, 6, 8 and 10 Gy.

Correspondence: I. Yamada.
Received 5 October 1989; and in revised form 14 February 1990.

### *Effects of L-dopa on radiosensitivity*

Two × $10^5$ cells were inoculated into 60-mm Petri dishes and incubated for 48 hours. One hundred $\mu g$ of L-dopa per millilitre was added to the culture medium immediately before irradiation. The cells were then irradiated with graded doses and incubated for 4 h at 37°C.

### *Effects of L-dopa on potentially lethal damage (PLD) repair*

For PLD repair studies, 2 × $10^5$ cells were inoculated into 60-mm Petri dishes and grown to confluence. During this period, the medium was changed on alternate days. Cells in the confluent state were irradiated with graded doses and incubated for 6 h at 37°C. To examine the effect of L-dopa on the PLD repair, 100 $\mu g$ of L-dopa per millilitre was added to the culture medium immediately before irradiation and similarly incubated for 6 h.

### *Colony formation*

After irradiation and L-dopa exposure, the cells of each treated group were washed and trypsinised, and an appropriate number of cells were plated in duplicate 60 mm Petri dishes containing 5 ml of the complete medium. The dishes were incubated at 37°C in an atmosphere of 95% air/5% $CO_2$ for 14 days. The resulting colonies that contained more than 50 cells were counted and the survival fraction of each group was calculated in reference to the untreated control group. At least three replicate experiments were conducted for each treatment. The respective survival curves then were constructed by plotting the surviving fraction as a function of radiation dose. The slope of the linear portion of the survival curves was fitted by a least-squares linear regression analysis and the $D_0$, $D_q$ and $n$ values were calculated. Further, a linear quadratic analysis was carried out for these survival curves, and $\alpha$ and $\beta$ values were calculated.

### *Cell cycle analysis*

Cell cycle distributions of melanoma cells treated with L-dopa and radiation were determined from DNA histograms measured by flow cytometry. Exponentially growing cells were exposed to 10 Gy radiation and then incubated with 100 $\mu g\ ml^{-1}$ L-dopa for four hours. The L-dopa was then removed by changing the medium. Twenty-four hours later, the cells were trypsinised from the dish. The cell suspension was washed twice with a phosphate buffered saline (PBS, pH 7.2), after which the cells (1 × $10^6$) in 1 ml of PBS were mixed with 3 ml of cold 95% ethanol, and incubated at −20°C for 60 min for fixation. The cells then were washed twice with PBS and incubated in a solution of 1 mg $ml^{-1}$ of ribonuclease (RNase A, 4396 U mg$^{-1}$, Worthington Biochemical Corp., Freehold, NJ, USA) in PBS for 30 min at room temperature. After enzyme treatment, the sample was mixed with 1 ml of 50 $\mu$g ml$^{-1}$ of propidium iodide (Calbiochem, San Diego, CA, USA) in PBS and kept at room temperature for 60 min for a DNA assay (Dean *et al.*, 1982). The DNA content per cell was assayed by flow cytometry, using a FACScan (Becton-Dickinson, Sunnyvale, CA, USA), with collection of fluorescence emissions having wavelengths longer than 590 nm. Some $10^5$ cells were analysed and the distribution histograms of the fluorescence intensity in linear scale were obtained. Cell cycle analysis by DNA distribution was performed by using the 'CCANA 1' program reported by Dean (1980), and the proportions in the $G_1$, S and $G_2$-M phases were calculated. Each data point represents the mean of three experiments. The same experiment and analysis were carried out for the untreated controls, the L-dopa only, and the radiation only groups.

### Results

### *Killing effects of L-dopa*

In order to examine the killing effects of L-dopa alone, exponentially growing cells were exposed to 100 $\mu$g ml$^{-1}$ of L-dopa for 0–6 hours and survival fractions were determined using a colony-forming assay. The plating efficiency of the untreated HMV-I cells was 78 ± 8%, and that of the HeLa S3 cells, 60 ± 7%. There was no significant reduction in the survival fraction in cells treated with L-dopa.

Next, HMV-I cells in the confluent state were exposed to 100 $\mu$g ml$^{-1}$ of L-dopa for 0–6 hours. The plating efficiency of the untreated HMV-I cells in the confluent state was 76 ± 3%, and there was no significant reduction in the survival fraction in cells treated with L-dopa.

### *Radiation sensitivities*

The radiation dose-response curve of the HMV-I and HeLa S3 cells are shown in Figure 1. Using a least-squares regression analysis to fit the survival curve, HMV-I cells had a $D_0$ of 1.49 ± 0.21 Gy, a $D_q$ of 2.92 ± 0.23 Gy, and an *n* of 7.0 ± 1.33. Thus, the survival curve of the HMV-I cells had a broader shoulder region than that of the HeLa S3 cells which showed a $D_0$ of 1.41 ± 0.13 Gy, a $D_q$ of 1.89 ± 0.18 Gy, and an *n* of 3.8 ± 1.20.

When the HMV-I cells were irradiated and then exposed to 100 $\mu$g ml$^{-1}$ of L-dopa for 4 h, there was significant reduction in the cell survival in each of the radiation doses examined, compared with the untreated cells ($P < 0.05$) (Figure 1). Further, L-dopa decreased the shoulder width of the survival curve considerably, as indicated by the values of a $D_0$ of 1.42 ± 0.11 Gy, a $D_q$ of 0.61 ± 0.24 Gy, and an *n* of 1.5 ± 1.12 (Table I). However, the same treatment with L-dopa did not significantly affect the radiation sensitivity of the HeLa S3 cells, i.e. a $D_0$ of 1.41 Gy ± 0.18, a $D_q$ of 1.90 ± 0.20 Gy, and an *n* of 4.0 ± 1.63.

### *PLD repair*

Figure 2 shows the survival curves of the HMV-I cells in the confluent state. When the HMV-I cells in the confluent state were incubated for 6 h after irradiation before replating, the repair of PLD was prominent; the ratio of the $D_0$ values before and after incubation being 1.7 (Table II). However, when L-dopa was added to the cell cultures immediately before irradiation and remained for 6 h before replating, PLD repair was significantly inhibited ($P < 0.01$).

### *Inhibition of cell cycle progression*

The DNA histograms shown in Figure 3 indicate that L-dopa alone did not produce significant changes in the cell cycle distribution compared to the untreated controls. An

**Figure 1** Survival curves of HMV-I and HeLa S3 cells after irradiation with or without L-dopa treatment. Two × $10^5$ cells were inoculated into 60-mm Falcon Petri dishes and incubated for 48 h. Cells were irradiated with graded doses and then exposed to 100 $\mu$g ml$^{-1}$ of L-dopa for 4 h at 37°C. Values represent a mean of three to five experiments; bars, s.e. ●, HMV-I after radiation alone; ○, HeLa S3 after radiation alone; ■, HMV-I after radiation and L-dopa; □, HeLa S3 after radiation and L-dopa.

**Table I** Radiobiological parameters of HMV-I human melanoma cells in the exponential state treated with L-dopa

| *Treatments* | *$D_0$ (Gy)* | *n* | *α ($Gy^{-1}$)* | *β ($Gy^{-2}$)* |
|---|---|---|---|---|
| Radiation | 1.49 ± 0.21 | 7.0 ± 1.33 | 0.070 ± 0.007 | 0.041 ± 0.009 |
| Radiation + L-dopa | 1.42 ± 0.11 | 1.5 ± 1.12 | 0.514 ± 0.012 | 0.011 ± 0.004 |

accumulation of $G_2$-M cells was noted in the irradiated cells. When L-dopa was combined with radiation, an additional increase in $G_2$-M cells was found. The percentages of cells in the $G_1$, S, and $G_2$-M phases after different treatments are listed in Table III.

### Discussion

Melanoma cells possess a unique metabolic pathway for the conversion of L-dopa to melanin that is said to be mediated by tyrosinase (Pawelek, 1976). Further, it has been shown that L-dopa is selectively incorporated by melanoma cells and that it exhibits a selective cytotoxicity (Wick *et al.*, 1977). Our present study has indicated that L-dopa potentiated radiation cytotoxicity towards human melanoma cells. The survival curve of exponentially growing HMV-I melanoma cells was modified by L-dopa treatment, and a decrease in the shoulder portion was especially prominent. Many investigators have demonstrated that the large shoulder in the survival curve of melanoma cells may be related to the poor radiation response that has been clinically observed in human melanomas (Barranco *et al.*, 1971; Fertil & Malaise, 1981). Furthermore, Sasaki (1987) has demonstrated that tumour

**Figure 2** PLD repair in HMV-I cells and its inhibition by L-dopa. Two × $10^5$ cells were inoculated into 60 mm Petri dishes and grown to confluence. Cells were irradiated with graded doses while in the confluent state. Either immediately after irradiation (●) or after 6 h incubation for PLD repair (○), the cells were trypsinised and seeded in duplicate 60-mm dishes for an assay of their colony-forming ability. For effects of L-dopa on the PLD repair, cells were incubated with 100 μg $ml^{-1}$ of L-dopa for 6 h after irradiation (■). Values represent a mean of three to five experiments; bars, s.e.

**Table II** Radiobiological parameters of HMV-I human melanoma cells in the confluent state treated with L-dopa

| *Treatments* | $D_0$ *(Gy)* | $D_0$ *ratio* | $\alpha$ *(Gy$^{-1}$)* | $\beta$ *(Gy$^{-2}$)* |
|---|---|---|---|---|
| Radiation (0 h) | 1.17 ± 0.23 | 1.0 | 0.175 ± 0.010 | 0.044 ± 0.010 |
| Radiation (6 h) | 1.97 ± 0.11 | 1.7 | 0.143 ± 0.007 | 0.026 ± 0.004 |
| Radiation + L-dopa (6 h) | 1.33 ± 0.21 | 1.1 | 0.209 ± 0.012 | 0.035 ± 0.005 |

cells showing a larger shoulder have remarkably high survival fraction (up to $10^9$-fold) following a course of fractionated radiotherapy (2 Gy per fraction, 30 fractions), when compared with tumour cells showing a smaller shoulder. Thus, the combined use of L-dopa may be an effective means of overcoming the large shoulder on the radiation survival curve seen in melanomas. Our data indicated that non-melanoma control cells were unaffected by the same L-dopa treatment. This suggests that L-dopa may potentiate the radiation toxicity selectively in melanoma cells, and probably does not affect normal tissue that has no tyrosinase. Thus, the therapeutic ratio in the management of melanomas may be enhanced by the use of L-dopa.

Our present data have also demonstrated that the PLD repair of the HMV-I melanoma cells was significantly inhibited by post-radiation incubation of the irradiated cells with L-dopa. Recently, radiotherapy using large dose per fraction has been proposed for the therapy of human melanoma (Habermalz & Fischer, 1976; Overgaard, 1980), aiming at overcoming the large shoulder in the melanoma

**Figure 3** DNA histograms of HMV-I cells after treatment with L-dopa and 10 Gy radiation. All measurements were made at 24 h after treatments. **a**, Untreated control; **b**, L-dopa only, **c**, radiation only; **d**, radiation and L-dopa.

cells. However, Weichselbaum and Little (1982*a*,*b*) have demonstrated that, the larger the fraction dose, the more prominent the repair of the PLD in the melanoma cells, and they have suggested that the greater repair of PLD may be another important factor determining the poor radiation response in melanomas. In the management of melanomas, therefore, L-dopa may also be an effective agent for inhibiting the PLD repair.

In addition, the enhancement of cell killing was found to be associated with increased blockage of the HMV-I cells by L-dopa in the $G_2$-M phase. Our data indicated that L-dopa exerted a similar degree of inhibition in cell cycle progression at different phases of the cell cycle, and that the HMV-I cells were most sensitive to radiation damage during the $G_2$-M phase. Thus, the increased blockage caused by L-dopa during the $G_2$-M phase may indicate increased cell damage and an inability to proliferate or, alternatively, may merely reflect the accumulation of dead cells during the $G_2$-M phase, and hence the potentiation of radiation cell killing by the L-dopa.

The biochemical mechanism of radiosensitisation by L-dopa remains to be studied. Wick *et al.* (1977) and Wick (1978) have postulated that L-dopa acts on melanoma cells through an initial conversion to quinones mediated by tryosinase and a subsequent scavenging action on the sulphydryl groups, by which DNA polymerase α may be inactivated. This hypothesis is supported by the fact that 1,2-benzoquinones have a marked affinity for DNA polymerase α (Graham *et al.*, 1978) and that L-dopa inhibits the activity of DNA polymerase α only in the presence of tyrosinase (Wick, 1980). Recently, Lonn and Lonn (1985) have demonstrated that DNA polymerase α is involved in the repair process of DNA lesions induced by X-ray irradiation in human melanoma cells. Hence, it might be possible that

**Table III** Percentages of HMV-I human melanoma cells at $G_1$, S and $G_2$-M phases of cell cycle measured 24 h after the completion of treatments

| | *Percentages* | | |
|---|---|---|---|
| *Treatments* | $G_1$ | *S* | $G_2$*-M* |
| Control | 59 ± 8 | 28 ± 6 | 15 ± 7 |
| L-dopa (100 μg $ml^{-1}$) | 58 ± 9 | 28 ± 8 | 14 ± 9 |
| Radiation (10 Gy) | 47 ± 5 | 25 ± 8 | 28 ± 2 |
| Radiation + L-dopa | 44 ± 7 | 18 ± 4 | 38 ± 3 |

L-dopa inhibits the repair of radiation-induced DNA lesions through inactivating DNA polymerase α, and thus potentiates the radiation cytotoxicity in melanoma cells.

This work has been supported in part by Grants-in-Aid for Cancer Research (60010031, 60010006, 61010004) from the Ministry of Education, Science and Culture of Japan.

### References

BARRANCO, S.C., ROMSDAHL, M.M. & HUMPHREY, R.M. (1971). The radiation response of human malignant melanoma cells grown *in vitro*. *Cancer Res.*, **31**, 830.

DEAN, P.N. (1980). A simplified method of DNA distribution analysis. *Cell Tissue Kinet.*, **13**, 299.

DEAN, P.N., GRAY, J.W. & DOLBEARE, F.A. (1982). The analysis and interpretation of DNA distribution measured by flow cytometry. *Cytometry*, **3**, 188.

FERTIL, B. & MALAISE, E.P. (1981). Inherent cellular radiosensitivity as a basic concept for human tumor radiotherapy. *Int. J. Radiat. Oncol. Biol. Phys.*, **7**, 621.

GRAHAM, D.G., TIFFANY, S.M. & VOGEL, F.S. (1978). The toxicity of melanin precursors. *J. Invest. Dermatol.*, **70**, 113.

HABERMALZ, H.J. & FISCHER, J.J. (1976). Radiation therapy of malignant melanoma: experience with high individual treatment doses. *Cancer*, **38**, 2258.

LONN, U. & LONN, S. (1985). Reduced repair of X-ray-induced DNA lesions in cells without functioning DNA polymerase α. *Radiat. Res.*, **102**, 71.

OVERGAARD, J. (1980). Radiation treatment of malignant melanoma. *Int. J. Radiat. Oncol. Biol. Phys.*, **6**, 41.

PAWELEK, J.M. (1976). Factors regulating growth and pigmentation of melanoma cells. *J. Invest. Dermatol.*, **66**, 1976.

SASAKI, T. (1987). Tumor progression and tumor stem cell heterogeneity in relation to tumor radioresistance. *Jap. J. Cancer Clin.*, **33**, 1560.

WEICHSELBAUM, R.R. & LITTLE, J.B. (1982*a*). Radioresistance in some human tumor cells conferred *in vitro* by repair of potentially lethal X-ray damage. *Radiology*, **145**, 511.

WEICHSELBAUM, R.R. & LITTLE, J.B. (1982*b*). The differential response of human tumours to fractionated radiation may be due to a post-irradiation repair process. *Br. J. Cancer*, **46**, 532.

WICK, M.M. (1978). Dopamine: a novel antitumor agent active against B-16 melanoma *in vivo*. *J. Invest. Dermatol.*, **71**, 163.

WICK, M.M. (1980). Levodopa and dopamine analogs as DNA polymerase inhibitors and antitumor agents in human melanoma. *Cancer Res.*, **40**, 1414.

WICK, M.M. (1983). The chemotherapy of malignant melanoma. *J. Invest. Dermatol.*, **80**, 61s.

WICK, M.M., BYERS, L. & FREI, E. III (1977). L-Dopa: selective toxicity for melanoma cells *in vitro*. *Science*, **197**, 468.

YAMADA, I., SEKI, S., MATSUBARA, O., ITO, S., SUZUKI, S. & KASUGA, T. (1987). The cytotoxicity of cysteinylcatechols and related compounds to human melanoma cells *in vitro*. *J. Invest. Dermatol.*, **88**, 538.